# Noninvasive electrical stimulation as a neuroprotective strategy in retinal diseases: a systematic review of preclinical studies

**DOI:** 10.1186/s12967-023-04766-4

**Published:** 2024-01-06

**Authors:** Jiaxian Li, Wei Zhou, Lina Liang, Yamin Li, Kai Xu, Xiaoyu Li, Ziyang Huang, Yu Jin

**Affiliations:** Department of Eye Function Laboratory, Eye Hospital, China Academy of Chinese Medical Sciences, 33 Lugu Road, Shijingshan District, Beijing, 100040 People’s Republic of China

**Keywords:** Noninvasive electrical stimulation (NES), Transcorneal electrical stimulation (TES), Whole-eye electrical stimulation (WES), Transscleral electrical stimulation (TsES), Transpalpebral electrical stimulation (TpES), Transorbital electrical stimulation, Neuroprotection, Retina

## Abstract

**Background:**

Electrical activity has a crucial impact on the development and survival of neurons. Numerous recent studies have shown that noninvasive electrical stimulation (NES) has neuroprotective action in various retinal disorders.

**Objective:**

To systematically review the literature on in vivo studies and provide a comprehensive summary of the neuroprotective action and the mechanisms of NES on retinal disorders.

**Methods:**

Based on the PRISMA guideline, a systematic review was conducted in PubMed, Web of Science, Embase, Scopus and Cochrane Library to collect all relevant in vivo studies on “the role of NES on retinal diseases” published up until September 2023. Possible biases were identified with the adopted SYRCLE’s tool.

**Results:**

Of the 791 initially gathered studies, 21 articles met inclusion/exclusion criteria for full-text review. The results revealed the neuroprotective effect of NES (involved whole-eye, transcorneal, transscleral, transpalpebral, transorbital electrical stimulation) on different retinal diseases, including retinitis pigmentosa, retinal degeneration, high-intraocular pressure injury, traumatic optic neuropathy, nonarteritic ischemic optic neuropathy. NES could effectively delay degeneration and apoptosis of retinal neurons, preserve retinal structure and visual function with high security, and its mechanism of action might be related to promoting the secretion of neurotrophins and growth factors, decreasing inflammation, inhibiting apoptosis. The quality scores of included studies ranged from 5 to 8 points (a total of 10 points), according to SYRCLE’s risk of bias tool.

**Conclusion:**

This systematic review indicated that NES exerts neuroprotective effects on retinal disease models mainly through its neurotrophic, anti-inflammatory, and anti-apoptotic capabilities. To assess the efficacy of NES in a therapeutic setting, however, well-designed clinical trials are required in the future.

## Background

Since the eighteenth century, there has been great enthusiasm and curiosity in studying the effects of electrical currents inside the human body [1]. Electrical activity has a crucial impact on the development and survival of neurons [2–4], the depolarization of neurons has a trophic effect on their development [5, 6], electrical stimulation (ES) activates the motor neuron cell body, accelerates axon regeneration, and increases the secretion of beneficial cytokines such as brain-derived neurotrophic factor (BDNF) [7, 8]. Currently, transcranial ES technology has been widely used in the treatment of various diseases related to brain neurons, including cognitive impairment, schizophrenia, depression, dementia, Parkinson's disease, stroke, traumatic brain injury, multiple sclerosis, and fibromyalgia [9–15]. The primary role of ES in the brain is to alter the polarity of the neuronal membrane, leading to a subthreshold shift in membrane potentials at the resting state to hyperpolarization or depolarization [16]. Neural networks have a more selective response to current fields than single neurons, as current flows can interfere with the functional connection, synchronization, and oscillatory activity of various cortical and subcortical networks [17].

Vision is imaged in the brain, the retina and optic nerve, which receive light stimuli and convert information into neural impulses, transmit them to the brain, are important components of the visual pathway [18]. Published studies have confirmed that after ES, healthy volunteers experience phosphenes involving the visual cortex [19], along with observable alterations in visual functions like vision, visual field, and contrast sensitivity [20–22]. If ES can influence a healthy visual brain, it has the potential to restore damaged visual systems as well.

The inner surface of the eye is lined with a type of light-sensitive tissue called the retina, which is responsible for the initial stage of visual processing. The retina's complex structure and function render it vulnerable to alterations from any kind of pathological injury [23]. Photoreceptors are in charge of detecting various light wavelengths over a broad spectrum of brightness. As first-order neurons that convert light energy into visual signals, healthy photoreceptors are critical for vision. In the late stages of illness, the loss of photoreceptors quickly causes visual impairment and, ultimately, retinal remodeling since afferent secondary (bipolar cells) and tertiary (retinal ganglion cells) retinal neuron signals are lost [24–27]. Retinal degeneration (RD), including retinitis pigmentosa (RP) and age-related macular degeneration (AMD), typically shows these pathological alterations.

The health of retinal ganglion cells (RGCs), whose axons converge to form the optic nerve and provide the last circuit between retinal processing and higher-level visual processing in the midbrain and cortex, is another factor that influences vision in addition to photoreceptor health. Damaged RGCs prevent the midbrain from receiving visual information for processing and interpretation [28]. RGC injury often occurs in diseases such as glaucoma, anterior ischemic optic neuropathy, and traumatic optic neuropathy.

The current retinal neuroprotection strategies can be divided into (1) drugs targeting survival pathways, including anti-apoptotic agents such as tauroursodeoxycholic acid, steroids, dopamine-related therapies, as well as growth factors such as ciliary nerve trophic factor (CNTF) and BDNF; and (2) the rehabilitative methods that increase endogenous, including physical exercise and ES [28]. The advantage of the former is that drugs can target the retina to produce beneficial effects, but the challenge is how to effectively deliver interventions to the target tissue. Physical exercise rehabilitation is one of the latter, with a greater emphasis on neuroprotective effects on multiple systems, and it has not yet been fully investigated as a potential intervention for retinal neuroprotection.

Research on creating an ES treatment for numerous eye conditions has significantly increased in recent years. The therapeutic approach is based on electrical current stimulation of neurons along the visual pathway [29]. Retinal implants (retinal prostheses) are a type of invasive ES that use energy converters to generate electricity to mimic photoreceptor activities [30]. Retinal prostheses can be implanted in three locations. Epiretinal prostheses are anchored to the retinal inner surface and electrically target the ganglion cell layer (GCL). Subretinal prostheses are inserted between the retina and the retinal pigment epithelial layer, primarily targeting the retinal inner nuclear layer. Suprachoroidal prostheses are placed between the choroid and the sclera to stimulate the retina from the outside [31]. The retinal implants provide an innovative method for restoring vision in degenerative retinal diseases. However, several limitations hinder their clinical advancement, such as the choice of implant materials, subpar visual quality, and constrained viewing angles. Especially as an invasive therapy, it carries the risk of serious complications [32].

In contrast to invasive ES, noninvasive electrical stimulation (NES) through the eyelids, orbit, and cornea has the benefit of minimal invasion, only touching the skin and cornea, with only mild adverse reactions reported, and may have a significant protective effect on the retina. For instance, transcorneal electrical stimulation (TES) is a non-invasive neuromodulatory method with positive effects on the evocation of visual cortical responses [33]. There is convincing evidence that TES can alter rats' brain oscillations [34, 35], and molecular evidence demonstrates that TES can stimulate non-visual brain regions as well [36]. The above favorable characteristics explain why it has been studied as a potential protective technique and is widely used in many diseases.

Our current study aimed to (1) evaluate reports on the neuroprotective effect of noninvasive electrical stimulation on in vivo models of retinal disorders through a systematic literature review, and (2) elucidate its potential mechanisms of action.

## Methods

The current systematic review was designed according to the Preferred Reporting Items for Systematic Reviews and Meta-Analyses (PRISMA) [37]. Two independent researchers participated in the systematic review at each stage (study search and selection, data extraction, and risk of bias appraisal).

### Searching strategy

Web of Science, PubMed, Embase, Scopus, and the Cochrane Library were all searched. Two authors independently searched all original papers that had been published up until September, 2023. Only articles in the English language were taken into consideration due to a language restriction for the selection. A combination of medical subject headings (MeSH) and free text terms were used to identify the diseases and interventions as follows:

- (i) retina OR retinitis pigmentosa OR retinal degeneration OR optic nerve OR retinal ganglion cell OR photoreceptor OR retinal neuroprotective OR retinal neuron
  AND
- (ii) noninvasive OR transcorneal OR transscleral OR transeyelid OR transorbital OR transpalpebral.
  AND
- (iii) electrical stimulation OR electric stimulation.

The generated reference lists were manually reviewed to find any potential research that the electronic searches had neglected. All the articles from these searches were exported to EndNote X8 with duplicate records deleted, as well as articles that were not part of in vivo studies. Articles were first screened by reading titles and abstracts, and those that were irrelevant or lacked complete text were excluded. The remaining articles were then screened based on the inclusion and exclusion criteria by reading the full text.

### Inclusion and exclusion criteria

The inclusion criteria were considered: (1) animal studies; (2) studies that focused on the effects and action mechanisms of NES on retinal diseases; (3) independent and full-text accessible original data.

The exclusion criteria were considered: (1) studies focused on electrical stimulation other than NES; (2) studies that analyzed the NES effect along with other treatments without isolated eyes for NES; (3) absence of control (the control had to be comparable to the eyes treated with NES).

### Data extraction and management

Two investigators performed data collection independently using an Excel sheet. Discrepancies were assessed by consensus, and when they were not initially reached, the third reviewer was consulted. The following information was extracted from each study: study title, author, year of publication, diseases, animal’s species, sex and age, types of animal model, number of animals per group (and number of animals in total if specified), routes and parameters, frequency and duration, time points, tissues studied, laboratory techniques, and major findings.

### Methodological quality appraisal for included studies

The methodological quality of the included studies was assessed using the SYRCLE’s risk of bias (RoB) tool, a RoB tool for animal intervention studies presented by the Systematic Review Centre for Laboratory Animal Experimentation (SYRCLE) [38]. It consists of ten items within six main domains, namely selection bias, performance bias, detection bias, attrition bias, reporting bias, and other sources of bias. The answer for the judgment of bias was either “YES” to indicate a low risk of bias, “NO” to indicate a high risk of bias, or “NC” to indicate an uncertain level of bias because of insufficient information. The items judged as “YES” were scored one point, and the scores of 10 items were added together for the quality score of each study.

## Results

### Study inclusion

**Fig. 1:**
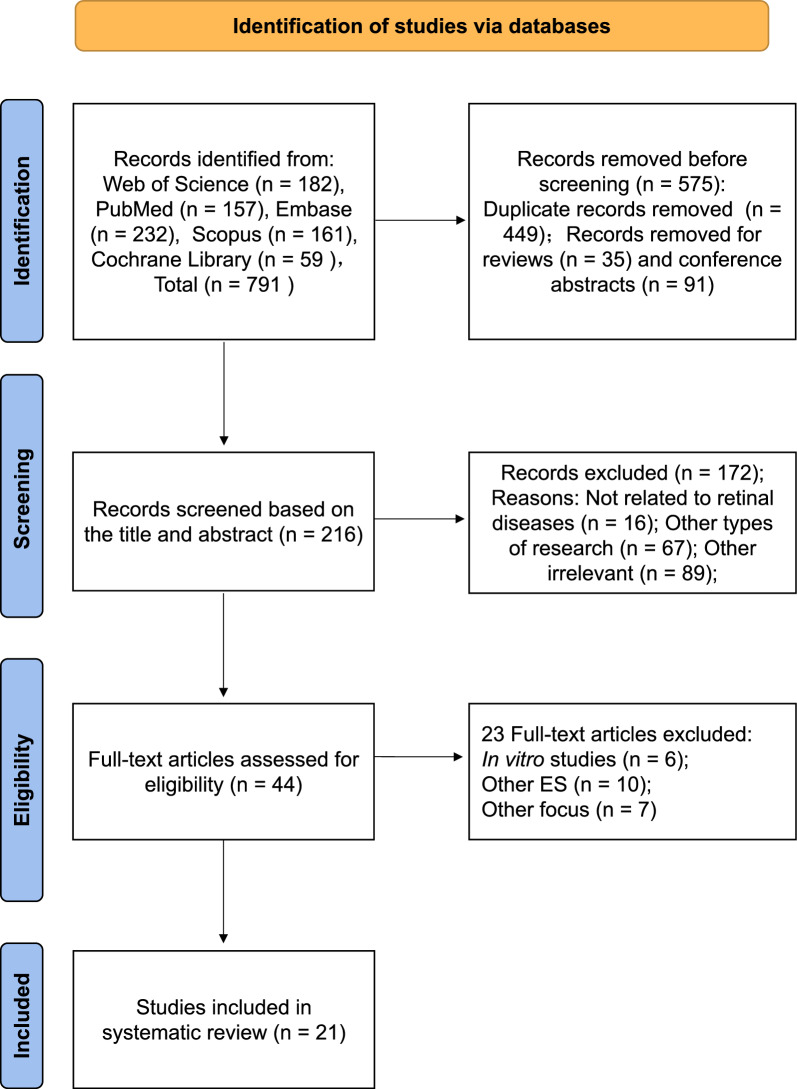
Flow chart of the results according to the search strategies

A total of 791 articles were extracted from the original retrieval, of which 182 articles appeared in Web of Science, 157 in PubMed, 232 in Embase, 161 in Scopus, and 59 in the Cochrane Library. Next, search filters were implemented, which excluded 575 articles (449 duplicates, 35 reviews, and 91 conference abstracts). By reading the titles and abstracts, 16 studies unrelated to retinal diseases, 67 other types of studies, and 89 other irrelevant studies were excluded. Thus, 44 articles were read in their full text. After analyzing these articles, 23 articles failed for at least 1 criterion and were excluded (in vitro studies, other electrical stimulation other than NES, and other focus). Finally, 21 articles were included in the systematic review [39–59]. The process and results are summarized in Fig. 1.

### Study characteristics

Included studies analyzed NES actions on different retinal injuries: retinal degeneration diseases (9 studies, including 6 studies themed on RP, 3 studies themed on RD); high-intraocular pressure injury (H-IOP, 3 studies); traumatic optic neuropathy: optic nerve crush (ONC, 5 studies), optic nerve transection (ONT, 3 studies); nonarteritic ischemic optic neuropathy (NAION, 1 study).

Among all the included studies, transcorneal electrical stimulation was used most frequently (17 studies). The other 4 studies involved whole-eye electrical stimulation (WES), transscleral electrical stimulation (TsES), transpalpebral electrical stimulation (TpES), and transorbital electrical stimulation, respectively. The majority of research only used one eye for treatment (15 studies). The most frequent parameters for ES were stimulation frequency of 20 Hz (16 studies), the rest ranging from 0.5 to 200 Hz (PPS); pulse duration of 1 ms/phase (9 studies), the rest ranging from 50 μs to 10 ms/phase; the current intensity of 100 μA (13 studies), the rest ranging from 4 to 700 μA; time duration of 1 h (13 studies), the rest ranging from 30 s to 6 h. The minimum treatment was only stimulated once, while the maximum was twice a week, lasting for 20 weeks.

The studies were conducted on laboratory animals, which included Sprague–Dawley (SD) rats (5 studies), Wistar rats (3 studies), Hooded rats (3 studies), Long-Evans rats (1 study), P23H-1 rhodopsin mutation rats (1 study), Royal College of Surgeons (RCS) rats (1 study), Rd10 mutant mice (2 studies), C57/BL mice, Rhodopsin knockout mice (Rho^−/−^) mice, DBA/2J mice, B6. Cg-Tg (Thy1-YFP) HJrs/J transgenic mice, Mongolian gerbils, and Rhodopsin P347L transgenic rabbits (1 study each). One of the studies used both Thy1-YFP mice and Hooded rats.

Animal models of RD (9 studies, including RP) were induced by different methods, six of which used different types of genetically engineered animals, including RCS and P23H-1 rats, Rd10 mice, Rho^−/−^ mice, and P347L rabbits; two of these studies used SD rats to establish the model induced by light damage (2500–16000 lx); another study established the model by intraperitoneal injection of *N*-methyl-*N*-nitrosourea (MNU) at 60 mg/kg (C57BL mice). Anterior chamber perfusion (NaCl solution) was used to establish H-IOP injury models (2 studies), and another study chose DBA/2J mice as the glaucoma model. Calibrated forceps crushed optic nerve surgery (0.1 mm jaw gap, 30 s) was used to establish the ON trauma model in Wistar, Hooded, and Long-Evans rats, optic nerve transection was another method (Wistar and SD rats). In addition, NAION in SD rats was induced by Rose bengal (RB)-laser induction.

The laboratory techniques involved in the included articles can be divided into three categories. Electroretinogram (ERG, 10 studies), multi-electrode-array (MEA, 2 studies), visually evoked potentials (VEP, 2 studies), electroencephalography (EEG, ECoG, 2 studies), vision-test (VIST), optokinetic tracking (OKT), and black-and-white transition box were utilized for visual function testing. Immunohistochemistry (IHC, 11 studies) and immunohistofluorescence (IF), Fluorogold (FG, 7 studies), and Oregon Green BAPTA retrograde labeling, hematoxylin–eosin stain (HE, 4 studies), toluidine blue stain (2 studies), terminal-deoxynucleotidyl transferase-mediated nick end labeling (TUNEL, 2 studies), anterograde labeling (2 studies), in vivo confocal neuroimaging (ICON, 3 studies), and confocal scanning laser ophthalmoscope (CSLO) were utilized for observing the histopathology and morphology of retinas. Western blot (WB, 6 studies), quantitative real-time PCR (qRT-PCR, RT-PCR, or qPCR, 6 studies), and Northern blot were utilized for molecular biology assays. Details are summarized in Table 1.

**Table 1:** Included studies on noninvasive electrical stimulation effects in animals with retinal disorder

Author, year (country)	Focus	Animals (sex and age)	Models	Sample size (*n*)	Routes and parameters	Frequency and duration	Time points	Tissues	Laboratory techniques	Major findings
Takeshi Morimoto, 2007 (Japan) [39]	RP	RCS rats (rdy/rdy) (sex not specified, 3 weeks old)	RCS rats	TES: *n* = 6, sham: *n* = 6 per group	TES: contact lens electrode; biphasic rectangular current pulses; 20 Hz; 0 (sham), 50, and 100 μA; 1 ms/phase; 1 h; left eye	Once a week; 3–9 weeks of age	Two, 4, 6 weeks after start of treatment	Eye ball	ERG, toluidine blue stain	The mean thickness of the ONL at 5, 7, and 9 weeks of age was thicker in eyes treated with TES of 100 μA, and the retinal function up to 7 weeks of age was preserved in RP rats
Hanif, 2016 (USA) [40]	RP	P23H-1 rats (sex not specified, neonatal)	P23H-1 mutant rats	WES: *n* = 10*,* sham: *n* = 15	Whole-eye ES: silver (Ag/AgCl) pellet electrode; sine wave current (4 μA peak to peak at 5 Hz); 30 min; single eye	Twice a week, 4–24 weeks of age	Four, 8, 12, 17, and 20 weeks after start of treatment	Eye ball	OKT, ERG, toluidine blue stain, RT-PCR	(1) WES increased the spatial frequency thresholds, improved the amplitude of OPs waves in ERG, and preserved the number of ganglion cells in RP rats; (2) One hour post-WES, BDNF, caspase 3, FGF-2, and GS increased at gene levels
Feng Liu, 2022 (China) [41]	RP	Rd10 mice (both sexes, neonatal)	Rd10 mutant mice	TsES: *n* = 59 or *n* = 92 per group, sham: *n* = 165	Transscleral ES: gold pads electrode; bi-phasic electric pulse (square wave, 2.5 ms pulse width,1 ms inter-pulse-interval); 20 Hz; 0 (sham), 50, 100 μA; 1 ms; 30 min; left eye	Daily or every other day, P20—P25	Five days after start of treatment	Eye ball	MEA, black-and-white transition box, IHC	(1) TsES modified the retinal light responses and reduced the high spontaneous firing of retinal ganglion cells; (2) 100 µA of TsES increased the light sensitivities of ganglion cells as well as their signal-to-noise ratios, and improved the survival of photoreceptors in RP mice
Ying-qin Ni, 2009 (China) [42]	RD	SD rats (male, adult, 225–250 g)	Exposure to bright blue light with 2500 lx for 24 h	TES: *n* = 6 or *n* = 2 per group, control: *n* = 6	TES: contact lens electrode; biphasic rectangular current pulses; 20–100 Hz; 100–500 μA; 3 ms; 1.5 h or 20 Hz; 200, 300 μA; 3 ms; 1 h; both eyes	Once before exposure or every 3 days after exposure, up to the 14th day	Seven, 14 days after light exposure	Eye ball	ERG, HE stain, IHC, qRT‐PCR, WB	(1) Both pre- and post-TES ameliorated the progressive photoreceptor degeneration, with the latter showing a relatively better and longer-term protective effect; (2) An upregulation of Bcl-2, CNTF, and BDNF and a downregulation of Bax in the retinas after TES, Bcl-2 and CNTF were selectively upregulated in Müller cells
Takeshi Morimoto, 2012 (Japan) [43]	RP	Transgenic rabbits (sex not specified, 6 weeks old)	Rhodopsin P347L transgenic rabbits	TES and sham: *n* = 5	TES: contact lens electrode; biphasic rectangular current pulses; 20 Hz; 700 μA; 1 h; left eye	Once a week, 6–12 weeks of age	Six weeks after start of treatment	Eye ball	ERG, IHC	(1) TES improved the a- and b-wave amplitudes of the photopic ERG and the b-wave amplitudes of the scotopic ERG; (2) TES preserved the thickness of ONL and promoted photoreceptor survival in the RP retinas
Andreas Schatz, 2012 (Germany, Switzerland, Brazil) [44]	RD	SD rats (sex and age not specified, 210.19 ± 14.63 g)	Exposure to bright light with 16,000 lx	TES and sham: *n* = 15, control: *n* = 3	TES: DTL electrodes; biphasic rectangular current pulses; 20 Hz; 200 μA; 10 ms/phase; 1 h; right eye	Once, 2 h before light exposure	Thirty-six hours, 7, 14, 21 days after light exposure	Eye ball	ERG, HE stain, IHC, TUNEL assay	(1) One week after light exposure, TES increased the Vmax of the retinas, and lowed the b-wave implicit time for the rod response after 2 weeks of light damage; (2) TES preserved a complete outer nuclear layer thickness, reduced photoreceptor cell death, and preserved outer segment length
Tao Ye, 2016 (China) [45]	RP	C57/BL mice (both sexes, 8–9 weeks old)	MNU (60 mg/kg body weight single, ip)	TES, sham and control: *n* = 50 per group	TES: contact lens electrode; biphasic rectangular current pulses; 20 Hz; 0(sham), 100, 200 μA; 1 h; both eyes	Days 1,3,6 after MNU injection	Eight days after start of treatment	Eye ball and whole retina	ERG, MEA, HE stain, IHC, qRT‐PCR	(1) TES increased the amplitudes of ERG b-wave. There were different rescuing kinetics existed among regional photoreceptors, the central region is more easily preserved than other regions; (2) The signal-to-noise ratio of TES-treated mice increased, indicating that the RGCs could transmit visual signals much more reliably; (3) Apoptosis factors Bax, Bcl-2, Calpain-2, neurotrophin BDNF, CNTF were involved in the protective effect of TES
Honghua Yu, 2020 (USA, Norway, China, The Netherlands) [46]	RP	Rho^−/−^ mice; (sex not specified, 6 weeks of age)	Rhodopsin knockout mice	TpES: *n* = 6 or *n* = 12 per group, control: *n* = 6, sham: *n* = 6	TpES: portable electrode probe; monophasic rectangular pulse; 2—200 PPS; 100 μA; random left or right eyes	Seven consecutive days (week 1) or 7 consecutive days every other week (weeks 1 and 3)	Before and 1, 2, 3, 4 weeks after start of treatment	Eye ball	ERG, IHC, TUNEL assay, qPCR	(1) TpES in Rho^−/−^ mice improved photoreceptor survival and electroretinography function; (2) TpES triggered residential retinal progenitor-like cells such as Müller cells to reenter the cell cycle, possibly producing new photoreceptors; (3) TpES directly stimulated cell proliferation and the expression of progenitor cell markers in Müller cells cultures, at least partially through bFGF signaling
Agadagba, 2022 (China, Egypt) [47]	RD	Rd10 mice (both sexes, P60-P90)	Rd10 mutant mice	TES: *n* = 6 per group, sham: *n* = 10	TES: silver wire electrode; biphasic square-wave pulses; 10 Hz; 400, 500, 600 μA; 2 ms/phase; 30 min; right eye	Once a day, repeated for 7 days	One day, 1, 2 weeks after start of treatment	*/*	ECoG	In retinal degeneration mice, the application of electrical stimulation to the retina clearly neuromodulates brain coherence and connection of visual and non-visual cortices, and the observed modifications are largely preserved
Xin Wang, 2011 (China) [48]	H-IOP	SD rats (famale, adult, 220–250 g)	Left eye, retinal ischemia by elevated IOP (IOP 120 mm Hg, maintained 60 min)	TES, control and sham: *n* = 3–7 per group	TES: contact lens electrode; biphasic rectangular current pulses; 20 Hz; 0 (sham), 300 μA; 3 ms/phase; 1 h; left eye	Once every 2 days until day 14, after ischemia	Six and 24 h, 3, 7, and 14 days after start of treatment	Eye ball and whole retina	FG retrograde labeling, ERG, HE stain, IHC, WB	(1) TES increased the average density of RGCs in retinas and better preserved the mean thickness of separate retinal layers; (2) TES preserved the ERG b-wave amplitude on day 7 after ischemia and recovered robustly on day 14; (3) The neuroprotective effect of TES is associated with an increase in GS levels in Müller cells
Lin Fu, 2018 (China) [49]	H-IOP	Mongolian gerbils (sex not specified, 3–5 months old)	Right eye, acute ocular hypertensive (IOP maintained 60 min)	TES, control and sham: *n* = 3–10 per group	TES: contact lens electrode; bipolar rectangular current; 20 Hz; 100 μA; 1 ms/phase; 1 h; right eye	Twice (day 1, day 4) every other week after IOP elevation, for 1 month	One, 4 weeks after start of treatment	Whole retina	ERG, IHC, WB, RT-PCR	(1) TES improved RGC survival and preserved the b-wave and PhNR amplitudes of ERG; (2) TES-treated eyes showed an increase in IL-10 expression, with a corresponding decrease in IL-6 and COX-2 expression as well as a decrease in NF-κB phosphorylation, which was related to suppression in microglial cell activation
Assraa Hassan Jassim, 2021 (USA) [50]	H-IOP	DBA/2J (D2) mice (both sexes, 3–5 or 10 months old)	Glaucoma model of DBA/2J (D2) mouse strain	TES: *n* = 18, control: *n* = 8, non-TES: *n* = 25	TES: contact lens electrodes; symmetric biphasic square wave; 20 Hz; 100 μA; 1 ms/phase; 10 min; both eyes	Once every 3 days for 8 weeks	Eight weeks after start of treatment	Whole retina	FG retrograde labeling, IHC, WB	TES resulted in RGC axon protection, a reduction in inflammatory cells and their activation (by inhibiting microglia activation and T cell infiltration), improved energy homeostasis (by reducing the pAMPK/AMPK ratio), and a reduction of the cell death-associated p75^NTR^
Ken-Ichiro Miyake, 2007 (Japan) [51]	ONC	Long-Evans rats (sex not specified, P100)	Left optic nerve crush (0.02 N, 5 s)	TES and sham: *n* = 4 or 5 per group	TES: contact lens electrode; biphasic square wave pulses; 20 Hz; 500 μA; 50 μs; 6 h; left eye	Once, after the post-crush VEP recording	Before, immediately after, 6 h, and 1 week after ONC	Eye ball	VEP, fluorescent anterograde tracer labeling	(1) TES immediately increased VEP amplitude impaired by ONC, and this augmentation was often preserved after 1 week; (2) After TES, a larger amount of retinal axons projected centrally beyond the crushed region, indicating that it protected retinal axons from the ensuing degeneration
Yuichi Tagami, 2009 (Japan) [52]	ONC	Wistar rats (male, adult, 250–300 g)	Left optic nerve crush (10 s)	TES: *n* = 5–10 per group, sham: *n* = 10, non-TES: *n* = 13	TES: contact electrode; biphasic rectangular current pulses; 20 Hz; 100 μA; 1 ms; 1 h; left eye	Once immediately after ONC (day 0); twice (days 0, 7); four times (days 0, 4, 7, 10); daily (days 0–12)	Three, 7, 12 days after ONC	Eye ball	Anterograde labeling, FG retrograde labeling, IHC	In retinas treated daily with TES, the mean number of regenerating axons significantly increased at 250 μm distal from the lesion, increased IGF-1 immunoreactivity was observed, and the survival of RGCs was enhanced
Petra Henrich-Noack, 2013 (Germany) [53]	ONC	Hooded rats (male, 7 weeks of age)	Bilateral optic nerve crush (0.1 mm jaw gap, 30 s)	TES: *n* = 12–13 per group, sham: *n* = 10–12 per group, non-TES: *n* = 8	TES: 3 mm diameter gold ring electrode; 100 μA; 1 ms; 30 s; different frequencies in order: 10–12-9–11-8–10- 9–12 Hz; both eyes	Immediately after and on days 3, 7, 11, 15, 19, 23 after ONC, frequencies in order with 5 s breaks, repeated once after a 2 min pause, 8 min of total stimulation time for one eye	During 42 days before and 43 days after ONC	–	VIST, ICON, EEG	TES induced long-term neuronal protection from delayed retrograde cell death, but in this case of severe axonal damage TES did not influence functional restoration and EEG signals recorded over the visual cortex
Petra Henrich-Noack, 2013 (Germany) [54]	ONC	Hooded rats (male, 6–7 weeks of age)	Bilateral optic nerve crush (0.1 mm jaw gap, 30 s)	TES and sham: *n* = 8 per group	TES: 3 mm diameter gold ring electrode; biphasic square-wave pulses; 20 Hz; 100 μA; 1 ms; 1 h; single eye	Twice (immediately after and day 11 after ONC)	Four days before and 3, 7, 11, 15 days after ONC	–	ICON	(1) TES delayed cell death after ONC, and RGC survival rate decreased over time; (2) TES reduced ONC-associated neuronal swelling and shrinkage, maintained cell morphology, especially in RGCs which survived long-term
Petra Henrich-Noack, 2017 (Germany,China, USA) [55]	ONC	(1) Hooded rats (male, 6–7 weeks of age); (2) B6.Cg-Tg (Thy1-YFP) HJrs/J transgenic mice (sex and age not specified)	(1) Rats: bilateral optic nerve crush (0.1 mm jaw gap, 30 s); (2) Mice: bilateral optic nerve crush (5 s)	(1) Rats: ES: *n* = 11, sham: *n* = 8, and control: *n* = 9; (2) Mice: ES and ES-control: *n* = 9, sham: *n* = 9	(1) Rats: transorbital ES; 3 mm diameter gold ring electrode; biphasic square-wave pulses (200 μA; 10 ms; different frequencies in order: 2–3-4–5-6–7-8- 6–5-4–3- 2 Hz; 23 min; both eyes); (2) Mice: transorbital ES; 2 mm diameter gold ring electrode; biphasic square-wave pulses; (100 μA; 1 ms; different frequencies in order: 10–12-9–11-8–10-9–12 Hz; 24 min; both eyes)	(1) Rats: days 0, 4 after ONC; (2) Mice: days 0, 3, 6, 9, 12 after ONC	(1) Rats: 21, 14, 10 days before and 4, 7, 18 days after ONC; (2) Mice: 8 days before and 3, 7, 14 days after ONC	(1) Rats: eye ball and whole retina; (2) Mice: /	(1) Rats:Oregon Green BAPTA retrograde labeling, ICON, VEP, luxol-fast-blue stain; (2) Mice: CSLO	(1) ES-induced dendritic pruning in surviving neurons in the initial post-ONC period; (2) Complete dendritic stripping following ES protects neurons from excitotoxic cell death by silencing them
Takeshi Morimoto, 2005 (Japan) [56]	ONT	Wistar rats (male, adult, 230–270 g)	Left optic nerve transection	TES: *n* = 6 per group, control: *n* = 12, non-ES: *n* = 8, sham: *n* = 6	TES: bipolar contact lens electrode; biphasic rectangular current pulses; 20 Hz; 100 μA; 0(sham)-3 ms/phase; 1 h; left eye	Once, commenced immediately after ONT	One hour to 14 days after start of treatment	Eye ball and whole retina	FG retrograde labeling, RT—PCR, Northern blot, WB, IHC	(1) TES rescued the axotomized RGCs by increasing the level of IGF-1 production by Müller cells; (2) IGF-1 immunoreactivity was originally localized in the Müller cell endfeet and then spread across the inner retina
Takeshi Morimoto, 2010 (Japan) [57]	ONT	Wistar rats (male, adult, 230–270 g)	Left optic nerve transection	TES: *n* = 6 per group, control: *n* = 12, non-ES: *n* = 8, sham: *n* = 6	TES: bipolar contact lens electrode; biphasic square pulses; (1) 0.5, 1, 2, 3, and 5 ms/phase, 100 μA, 20 Hz, 60 min; (2) 50, 100, 200, 300 and 500 μA, 1 ms/phase, 20 Hz, 60 min; (3) 0.5, 1, 5, 20, 50, and 100 Hz, 100 μA, 1 ms/phase, 60 min; (4) 15, 30, and 60 min, 100 μA, 1 ms/phase, 20 Hz; (5) 100 μA, 1 ms/phase, 20 Hz, 60 min; left eye	Once or four times (day 0, 4, 7, and 10) after ONT	Seven or 14 days after start of treatment	Whole retina	FG retrograde labeling	(1) Histologically, the optimal neuroprotective parameters for TES were pulse duration of 1 and 2 ms/phase, current intensity of 100 and 200 μA, stimulation frequency of 1, 5, and 20 Hz; (2) More than 30 min of TES was necessary to have a neuroprotective effect; (3) Symmetric pulses without an inter-pulse interval were most effective; (4) Repeated ES was more neuroprotective than a single ES
Houmin Yin, 2016 (China) [58]	ONT	SD rats (male, adult, 220–250 g)	Right optic nerve transection	TES, control and sham:* n* = 5 per group	TES: gold electrode; biphasic rectangular current pulses; 20 Hz; 0 (sham), 200 μA; 1 h; right eye	Days 0 and 4, or days 0, 4, 7 and 10 after ONT	Seven, 14 days after ONT	Whole retina	FG retrograde labeling, IF, WB	TES promoted RGC survival after ONT accompanied by reduced microglial activation and microglia-derived TNF-α production
Takako Osako, 2013 (Japan) [59]	NAION	SD rats (male, age not specified, 220–250 g)	RB-laser induction: RB (2.5 mM, 1 mL/kg, tail vein), laser (514 nm laser, 500 μm, 12 s, photoactivation of ON)	TES: *n* = 7 or12 per group, control: *n* = 7 or 8 per group	TES: monopolar contact lens electrode; biphasic square pulses; 20 Hz; 100 μA; 3 ms/phase; 1 h	Days 1, 4, 7, 14, and 28 after induction	Fourteen, 28 days after start of treatment	Whole retina	ERG (STR), FG retrograde labeling	TES preserved the decreasing STR amplitude and the decreasing RGC numbers in NAION. It was effective for preserving decreasing RGC numbers and function in the chronic stage of NAION

### Methodological quality

SYRCLE’s tool was used to assess the risk of bias in animal experiments. The quality scores ranged from 5 to 8 points. Overall, regarding selective bias, 11 studies (52.38%) mentioned “randomization”, but did not introduce specific approaches, and the rest did not report sequence generation. While 17 studies (80.95%) reported comparable baseline characteristics between control and experimental groups, nevertheless, none study clarified if allocation was concealed. Therefore, selective bias is the main reason for the deduction of quality scores. Regarding performance bias, 14 studies (66.67%) made it clear that the animals were housed in identical feeding conditions, such as the same temperature, humidity, light levels, and reported blinding while performing the experiments. Regarding detection bias, random outcome assessment while performing the experiments was reported for only two studies, but blinding while assessing the outcomes was reported for all studies. Low-risk bias was captured for all studies in the incomplete outcome data and the selective outcome reporting item, although none of these studies reported protocols, this judgment was validated based on what was reported in the methods. Eighteen studies (85.71%) were considered to be low risk in the other bias item, but three studies were the opposite, they used one eye as the experimental eye and the other as the control, which could result in a high risk of bias. The bias risk of in vivo studies is summarized in Table 2.

**Table 2:** Bias risk of included studies

Study/Bias	Selection bias			Performance Bias		Detection Bias		Attrition Bias	Reporting Bias	Other Bias	Quality score(“YES” items)
	**Sequence generation**	**Baseline characteristics**	**Allocation concealment**	**Random Housing**	**Blinding**	**Random Outcome Assessment**	**Blinding**	**Incomplete Outcome Data**	**Selective Outcome Reporting**	**Other sources of Bias**	
Takeshi Morimoto, 2007 (Japan)	NO	NC	NC	YES	YES	NC	YES	YES	YES	NO	5
Adam M.Hanif, 2016 (USA)	NC	YES	NC	YES	YES	NC	YES	YES	YES	YES	7
Feng Liu, 2022 (China)	NC	NC	NC	YES	YES	NC	YES	YES	YES	YES	6
Ying-qin Ni, 2009 (China)	NC	YES	NC	YES	YES	NC	YES	YES	YES	YES	7
Takeshi Morimoto, 2012 (Japan)	NO	NC	NC	YES	YES	NC	YES	YES	YES	NO	5
Andreas Schatz, 2012 (Germany, Switzerland, Brazil)	NO	YES	NC	YES	YES	NC	YES	YES	YES	YES	7
Tao Ye, 2016 (China)	NC	YES	NC	YES	YES	NC	YES	YES	YES	YES	7
Honghua Yu, 2020 (USA, Norway, China, Netherlands)	NC	YES	NC	YES	YES	YES	YES	YES	YES	NO	7
Stephen K. Agadagba, 2022 (China, Egypt)	NO	YES	NC	NC	NC	NC	YES	YES	YES	YES	5
Xin Wang, 2011 (China)	NC	YES	NC	NC	NC	NC	YES	YES	YES	YES	5
Lin Fu, 2018 (China)	NO	NC	NC	YES	YES	NC	YES	YES	YES	YES	6
Assraa Hassan Jassim, 2021 (USA)	NC	YES	NC	YES	YES	YES	YES	YES	YES	YES	8
Ken-Ichiro Miyake, 2007 (Japan)	NO	YES	NC	NC	NC	NC	YES	YES	YES	YES	5
Yuichi Tagami, 2009 (Japan)	NO	YES	NC	NC	NC	NC	YES	YES	YES	YES	5
Petra Henrich-Noack, 2013 (Germany)	NO	YES	NC	YES	YES	NC	YES	YES	YES	YES	7
Petra Henrich-Noack, 2013 (Germany)	NC	YES	NC	YES	YES	NC	YES	YES	YES	YES	7
Petra Henrich-Noack, 2017 (Germany,China, USA)	NC	YES	NC	YES	YES	NC	YES	YES	YES	YES	7
Takeshi Morimoto, 2005 (Japan)	NO	YES	NC	NC	NC	NC	YES	YES	YES	YES	5
Takeshi Morimoto, 2010 (Japan)	NO	YES	NC	NC	NC	NC	YES	YES	YES	YES	5
Houmin Yin, 2016 (China)	NC	YES	NC	YES	YES	NC	YES	YES	YES	YES	7
Takako Osako, 2013 (Japan)	NC	YES	NC	NC	NC	NC	YES	YES	YES	YES	5

### NES effect on retinal and visual function

Thirteen studies provided functional evaluation of retinas after treatment with NES. NES not only improves retinal and visual function in assessments like electrophysiological analysis and functional testing, but it also affects the neurons of cerebral cortex, especially the visual cortex.

#### NES preserved the function of retinal cells

In different retinal degeneration models (including RP), NES exhibited varying degrees of protective effects on retinal and visual function. In RCS rats, the amplitude of ERG b-wave or STR-like negative responses was greater than that of eyes with sham stimulation in the TES-treated eyes [39]. However, in the late stage of retinal degeneration (at 9 weeks old), the mean thickness of outer nuclear layer (ONL) in the TES-treated eyes was still thicker, but there was no significant change in the amplitude of b-wave (by ERG). WES protected the visual function of P23H-1 rats. Hanif et al. [40] found that over a period of 17 weeks, the spatial frequency threshold of WES-treated eyes increased by about 18% in the first 4 weeks, and then maintained a threshold of about 11% higher than sham's eyes. In the following weeks 4–17, the average spatial frequency threshold ratio of WES rats increased by 7% to 18% (by OKT). Similarly, inner retinal function, as measured by ERG oscillatory potentials (OPs), showed improved OPs amplitudes at 8 and 12 weeks post-WES.

In rd10 mice [41], an MEA record was used to analyze the light response of photoreceptors, bipolar cells, and ganglion cells. Compared to the sham surgery, 100 µA of TsES increased the amplitudes of N1, N2, and P2 waves by 118%, 120%, and 127%, respectively. (the N1 and N2 waves arise from photoreceptors [60, 61] and P1, P3 waves arise from ON and OFF bipolar cells). TsES improved the light response of individual RGCs, which are output neurons that transmit visual signals from the retina to the brain, TsES mainly improved the signal-to-noise ratio and sensitivity of RGCs by reducing abnormally high self-discharge. Honghua Yu et al. [46] reported that TpES effectively improved retinal function in Rho^−/−^ mice, the marked increases in b-wave amplitudes of photopic Pho 600, 3-Hz, and 10-Hz flicker (a typical indicator of cone function) were detected in ES-treated eyes at 1, 2, and 3 weeks after the first ES. However, the effect of the 7-day ES treatment was temporary, adding an additional session for 7 consecutive days every other week of ES prolonged the benefit (by ERG). Moreover, the a- and b-wave amplitudes of the photopic ERG and the b-wave amplitudes of the scotopic ERG at higher stimulus intensities were larger in the TES eyes than in the sham eyes of Tg rabbits, indicating that TES preserved the cone components better than rod components, although in Tg rabbits the rod components are more affected than the cones [43].

Similar protective effects have been observed in the intense light exposure and MNU-induced models of RD. Post 14 days of light exposure, TES (200 μA, 300 μA) treatment significantly increased the rod photoreceptor a-wave amplitudes with stimulation intensities ranging from − 8 dB to 2.5 dB, while b-wave exhibited higher responses with stimulation intensities ranging from − 24 dB to 2.5 dB compared with the control group (scotopic ERG) [42]. Another study showed that one week after light exposure, the ERG Vmax of the TES-treated retinas was higher than that of the sham-treated retinas [44]. The b-wave implicit time for the rod response was lower in the TES-treated retinas compared with the sham retinas 2 weeks after light damage.

The degree and regions of protection provided by NES for retinal and visual function are related to the current intensity. In the MNU-induced RP mice [45], the photopic and scotopic ERG b-wave amplitudes of the retina treated with 100 and 200 μA TES were significantly increased, with the 200 μA TES having significantly higher amplitudes than the 100 μA TES. After quantifying topographic photoreceptor function of TES-treated retinas, it was found that compared with the normal control retinas, the central, mid-peripheral, and peripheral regions of the 200 μA TES-treated retinas retained 61.3%, 50.1%, and 41.8% of photoreceptor function, respectively. The retinas treated with 100 μA TES retained 50.8%, 39.8%, and 31.5% of photoreceptor function in these three regions, respectively (by MEA). The signal-to-noise ratio (SNR) was calculated to analyze the efficiency of visual signal transmission. In MNU-induced RP mice, the impaired light-induced response and spontaneous hyperactivity collectively contributed to decreased SNR values. RGCs in the 200 μA TES-treated retinas could transmit visual signals much more reliably and economically, due to the SNR value in the 200 μA retinas being at least twofold larger than that in the 100 μA retinas, and 16-fold larger than that in the sham retinas.

NES protects the retina from damage caused by high intraocular pressure. The TES-treated retinas had a 50.5% higher ERG b-wave amplitude and a 42.9% higher PhNR (the first trough following b-wave) amplitude compared to the sham-treated retinas at 1 week after H-IOP [49]. There was further improvement in b-wave and PhNR amplitudes, reported 61.8% and 44.1% higher than that in the sham-treated retinas at 1 month, respectively. In the ischemic rats model induced by H-IOP [48], the b-wave amplitudes of scotopic ERG were well preserved and recovered in the TES-treated retinas. It is worth noting that compared with the control retinas, the b-wave amplitude of TES-treated retinas immediately increased significantly, even higher than the normal retinas during the initial period of dark adaptation on day 7 and almost entire period of dark adaptation on day 14.

The optic nerve crush immediately attenuated the VEP amplitude. Ken-Ichiro Miyake et al. [51] found that TES augmented the VEP that had deteriorated due to the optic nerve crush. After TES, VEP amplitude significantly increased and was ~ 200% larger than that immediately after the crush. The recovery index of VEP in TES-treated eyes increased to 273% (6 h) and 179% (1 week) of the value after the crush (by VEP). In addition, TES protected the visual function of rats from NAION damage.

#### The effect of NES on cerebral cortex

Agadagba et al. [47] demonstrated that electrical stimulation of the retina affects not just the neurons in the primary visual cortex but also appears to activate rodent prefrontal cortical connection networks. Spontaneous ECoG was carried out in rd mice to investigate neuromodulation of functional and directional connectivity aspects in both visual and non-visual brain cortices after short- and long-term retinal electrical stimulation in retinal degeneration. The results showed that extended TES triggers a long-lasting improvement of coordinated theta, alpha, and beta waves in rd mice, which exhibits high levels of interregional coherence and connectivity as well as synchronized phase amplitude coupling characteristics between theta and gamma oscillations. This sustained improvement in phase amplitude coupling (PAC), coherence, and directional connectivity was seen in the non-visual region (prefrontal cortex) of stimulated animals as well as the visual region (primary visual cortex).

### NES effect on retinal histomorphology

Twenty studies observed the protective effect of NES on retinal histomorphology, which mainly involved photoreceptors, RGCs and other retinal cell components.

#### NES preserved photoreceptors

In different retinal disorder models, NES exhibited varying degrees and regions of protective effects on photoreceptor histomorphology. In rd10 mice [41], retinal degeneration resulted in the ONL being thin with only one layer of soma remaining. TsES improved the survival rate of rd10 photoreceptor cells, it increased the number of layers to 2–3, and the thickness of ONL from the center to the peripheral region slightly thickened at each location (by IHC). In Rho^−/−^ mice [46], TpES promoted photoreceptor survival, the ONL thickness and the number of cone cells were preserved after ES treatment (by IHC), and fewer TUNEL + apoptotic photoreceptors in ES-treated retinas were detected. Assessment for photoreceptor gene expression demonstrated higher levels, including recoverin, G-opsin, and B-opsin, in ES-treated retinas (by qPCR).

In Tg rabbits [43], the loss of photoreceptors was maximum in the visual streak, a band of acute vision across the retina where the photoreceptor density is highest, ONL was only found in a row of nuclei loosely arranged, and the loss of photoreceptors was not significantly different in other regions outside at 12 weeks of age. TES rescued photoreceptors in the visual streak, the number of rows of nuclei in the ONL was 2–3 rows; the nuclei were closely packed in the retina receiving TES, and the thickness of ONL increased, indicating that the neuroprotection of photoreceptors was limited to the visual streak (by IHC).

The degree and area of protection provided by NES to photoreceptors are related to current intensity. Compared with 50 μA, the survival of photoreceptors in RCS rats treated with 100 μA TES was more significant [39]. To determine whether the differences in the thickness of the ONL were localized or widespread across the retina, the mean thickness of the ONL was determined at 18 points along the superior–inferior plane of the eye. It was found that the mean ONL thickness at each point in the superior and inferior hemispheres of the retinas was significantly thicker than that of the control retina, indicating that the neuroprotective effect of TES on photoreceptors may extend throughout the entire retina with current (by toluidine blue stain).

Tao Ye et al. [45] observed similar results in mice. Since rod cells account for at least 96% of total photoreceptors in the mouse retina, ONL thickness mainly indicates rod integrity and could be considered an indicator of rod number and vitality. In MNU-induced RP mice, photoreceptors in the central region were more sensitive to TES treatment, compared with the normal control retinas, the central, mid-peripheral, and peripheral regions of the 200 μA TES-treated retinas retained 57.1%, 46.6%, and 31.7% of the ONL thickness, respectively. The retinas treated with 100 μA TES retained 38.1%, 33.0%, and 23.5% of the ONL thickness in these three regions, respectively (by HE stain). Besides the rods, TES treatment effectively saved cone cells. Compared with the normal control retinas, the 200 μA TES-treated retinas retained 55.6% of cone density, while the 100 μA TES-treated retinas only retained 36.6% (by IHC).

The TES start time had an impact on the degree of protection as well. After being exposed to intense light [44], photoreceptor cell death mainly occurred in the superior retina, the length of IS/OS (inner segment and outer segment), and the ONL thickness of photoreceptors were reduced. At 14 days, only one row of cells remained of the photoreceptors in the superior hemisphere's posterior retina, which were most vulnerable to light damage, and the average ONL thickness was only 34.24% of that of normal rats (by HE stain). TES-treated retinas showed a significant preservation of the IS/OS length at 3/4 positions in the superior retina and partly in the inferior retina, and the ONL thickness of whole retina was saved (by TUNEL, HE, and IHC). When compared to acute pre-TES, which only provided temporary protection against photoreceptor degeneration after 7 days, chronic and low-intensity post-TES dramatically enhanced photoreceptor survival up to 14 days following light exposure [42]. Additionally, peripheral retinas as well as the superior and inferior central retinas had a neuroprotective effect following TES (by HE stain).

#### NES preserved retinal ganglia cells

In different retinal disorder animal models, NES exhibited varying degrees of protective effects on retinal ganglia cells and their axons. In the P23H-1 RD rats [40], the nuclei density in the GCL was visibly increased in WES-treated retinas, cell density in the RGC layer from the two superior and two inferior 0.5 mm regions of the retinal cross sections increased by 17–39%. Similarly, the total cellular density in the RGC layer from all regions increased by 14% overall (by toluidine blue stain).

In the ischemic rats model induced by H-IOP [48], TES retained 75% of the RGCs equivalent to normal rat retinas on day 7 after ischemic injury, and it was still able to preserve 60% of the RGCs on day 14. As a comparison, the RGC density in the sham surgery group was only 49% of that of normal rats (by FG retrograde labeling). Similarly, HE staining showed that TES better preserved the mean thickness of separate retinal layers, including the inner limiting membrane to outer limiting membrane, inner plexiform layer, and ONL (by HE stain). Lin Fu et al. [49] found that in the TES-treated retinas, which underwent electrical stimulation twice weekly for the entire month, there was a 39.2% higher overall RGC density compared to the sham-treated retinas. The TES significantly ameliorated secondary cell death after the acute ocular hypertension (AOH) injury (by IHC). In the mouse model of glaucoma [50], it was found that more RGC axons survived in the eyes treated with TES.

In the early stages after optic nerve trauma, NES effectively preserved the morphology and survival of RGCs. RGC survival following an optic nerve crush was shown to be greatly improved when TES was used [54]. Early post-traumatic periods (day 3) revealed RGC death in untreated animals, while TES-treated retinas appear to be almost undamaged. This indicates that TES influences in the early phase of the pathophysiological process. ICON analysis of the soma size changes in TES-treated retinas early after axonal trauma showed the absence of the typical sequence of cell swelling and shrinkage expected after injury, demonstrating that TES has a significant impact on the post-traumatic pathophysiology. Additionally, in vivo imaging demonstrated that transorbital ES caused dendritic pruning in surviving neurons during the initial post-ONC period [55]. In contrast, dendrites in untreated retinas degenerated slowly after the axonal trauma and neurons died (by ICON). The hypothesis that cell signaling is eliminated in the remaining neurons was supported by the total loss of VEP. However, intracellular free calcium imaging revealed that the cells were still alive despite this indication of “silencing” (by Oregon Green BAPTA labeling). Therefore, early after trauma, complete dendritic stripping following transorbital ES protects neurons from excitotoxic cell death by silencing them.

TES would rescue the retinal axons from degeneration in addition to improving their functional recovery [51]. In TES-treated animals, many labeled fibers on the central side of the crushed region were found, a clear fluorescent signal was observed in both the lateral geniculate nucleus (LGN) and superior colliculus (SC), areas that are targets of the retinal axons, while these markers were not found in unstimulated animals. How much tracer had been transported beyond the crushed region was estimated by calculating the tracer transport index, which compares the fluorescence intensity on each side of the crushed region. The value of the index was significantly higher in the stimulated animals than in the unstimulated ones (by fluorescent anterograde tracer labeling).

Similar to photoreceptors, the degree of protection provided by NES to RGCs is related to the repetitions, current intensity, pulse duration, and stimulation frequency. Daily application of TES significantly promoted the survival of RGCs after the crush (by FG retrograde labeling). It promoted regeneration of RGC axons within a distance of 250 μm of the crush site, and the regeneration gradually increased as the number of TES applications increased (by anterograde labeling) [52]. TES promoted the survival of RGCs after ONT (by retrograde labeling) [58]. Retinas that had received TES had many more surviving RGCs than those without electrical stimulation [56]. The increase in the densities of RGCs depended on the pulse duration of electric current. TES of 0.5 ms/phase pulse duration significantly increased the number of RGCs (70% of the normal density). In addition, TES of 1- and 3-ms/phase pulse duration further increased the density up to 85% and 83%, respectively, of normal. The shapes of surviving RGCs were similar to those of the RGCs in the intact retinas (by FG retrograde labeling). Takeshi Morimoto et al. [57] confirmed that the optimal neuroprotective parameters for TES were pulse duration of 1 and 2 ms/phase, current intensity of 100 and 200 μA, stimulation frequency of 1, 5, and 20 Hz, more than 30 min of TES was necessary to have a neuroprotective effect, repeated ES was more neuroprotective than a single ES. Symmetric pulses without an inter-pulse interval were most effective (by FG retrograde labeling).

NES protected the survival of RGCs in pathology, however, this morphological protective effect did not match its demonstrated functional performance in some animal models. Petra Henrich-Noack et al. [53] revealed that ONC significantly decreased the number of RGCs at 4 weeks after the lesion, more RGCs had died and the percentage of surviving cells decreased to 8.6% compared to baseline. This cell loss was significantly less pronounced in the TES-treated rats, in which still 28.2% of the RGCs could be detected (by ICON). However, improved neuronal survival did not support recovery of visual function (VIST) nor allow EEG alterations. In the NAION rats [59], the decreased amplitude in the scotopic threshold responses (STR) of ERG of the TES group was better preserved than in the control group on the 28th day, not on the 14th day after induction, but RGC survival of the TES group was larger than in the control group on the 14th and 28th days. The above results mean that the preservation effect of TES for visual function could be slightly delayed compared with that for cell survival (by FG retrograde labeling).

#### NES effect on other retinal cells

The effects of NES on other retinal components were mainly concentrated on Müller cells and microglia cells. In Rho^−/−^ mice [46], Müller cells could be inducted to proliferate and migrate toward the ONL by NES, whereas in rare cases they were found to colocalize with the photoreceptor marker recoverin. Two days after ES, Müller cells exhibited significantly increased expression of neurogenic signals Sox2, Wnt1, Wnt3a, and Wnt7a, as well as photoreceptor progenitor cell markers Chx10, Crx, Nr2e3, and Nrl. Two weeks after ES, some Müller cells could be seen to develop typical photoreceptor morphology and express mature photoreceptor-specific marker recoverin. The numbers of cells expressing the photoreceptor and retinal neuron markers recoverin and βIII-tubulin in ES-treated cultures showed a significant increase compared to the sham (by IHC). Therefore, ES directly stimulates Müller cells to promote their progenitor cell potential and photoreceptor progeny.

In addition, one week after acute ocular hypertension, microglia density increased 3.3-fold in the sham-treated retinas compared with the normal retinas, but only 2.5-fold in the TES-treated retinas [49]. The microglia cell density was 1.34 folds higher in the sham-treated retinas compared to normal retinas at 1 month. Microglia activation was fully reduced in the TES-treated retinas, and density was identical to that of normal retinas (by IHC).

### Possible neuroprotective mechanisms of NES

Ten studies revealed the potential neuroprotective mechanisms of NES on retinal diseases, which mainly involved neuro-nutrition, anti-inflammatory, anti-apoptosis, and other effects.

#### NES effect on neurotrophins and growth factors

The neuroprotective effect of NES on the retina is closely related to its regulation of neurotrophins, including CNTF, BDNF, and basic fibroblast growth factor (bFGF). After TES (300 μA) [42], the mRNA levels of both neurotrophic factors CNTF and BDNF were up-regulated on the first day in the RD rats, CNTF peaked on the 7th day, and BDNF peaked on the 3rd day, both of their mRNAs decreased to baseline levels, at 14 days (by qRT-PCR). In which, the expression of CNTF showed a time-dependent and radial expanding pattern from the GCL to the outer retina, it was selectively upregulated in Müller cells (by IHC). Especially in the retinas treated with 200 μA, where the mRNA levels of BDNF and CNTF were higher than those in the retinas treated with 100 μA [45]. Such findings suggested that neurotrophic factors BDNF and CNTF were involved in the TES-induced protective effects and they were related to the current intensity (by qRT-PCR).

BDNF involves the preservation of retinal cells denatured by toxic light and ischemic damage. Fibroblast growth factor 2 (FGF-2), as a mediator for retinal preservation, was associated with upregulation of growth factor mosaicism. Hanif et al. [40] found that the gene expression levels of BDNF, and FGF-2 in the retina of P23H-1 rats increased after a sine wave current (4 μA peak to peak at 5 Hz) WES treatment (by RT-PCR). However, these changes in gene expression occur quickly, by 1 h post-WES, and are back to normal by 24 h post-WES. The survival of RGCs likewise depends on BDNF, phosphorylation of the tyrosine kinase receptor B (TrkB) is an indication of its activity and binding of BDNF. Assraa Hassan Jassim et al. [50] demonstrated that TES-treated retinas had significantly greater TrkB phosphorylation than control retinas. Meanwhile, TES reduced p75^NTR^ in glaucomatous retinas to a level similar to that of the healthy retinas (by WB). p75^NTR^ is dysregulated in glaucoma models and has been shown to induce neuronal apoptosis. Moreover, ES-induced retinal production of bFGF contributes to the enhanced proliferative and neurogenic potential of Müller cells (by qPCR) [46].

NES exerts neuroprotective effects by regulating the expression of insulin-like growth factor 1 (IGF-1), but it is related to treatment repetitions. In the GCL and the outer plexiform layer of the normal rat retina, IGF-1 immunoreactivity was barely detectable [52]. However, retinal IGF-1 was elevated in the entire retinal layer with daily TES until day 12, but this was not the case with a single application. The axonal regeneration by the daily TES was completely blocked by a specific antagonist to the IGF-1 receptor, whereas the promotion of RGC survival was not prevented (by IHC). RT-PCR analysis showed that the expression level increased for the mRNA of IGF-1 depending on the pulse duration of the TES [56]. The expression of IGF-1 mRNA in the retina with 1-ms/phase pulses of TES was higher than that with 0.5-ms/phase, and this difference was maintained for at least 1 week. The increase in IGF-1 expression achieved the same results in WB and Northern blot analyses.

In addition, Takeshi Morimoto et al. [56] showed that IGF-1 was located in the endfeet of the Müller cells, and TES activated the Müller cells to produce more IGF-1 and release it into the inner retina. Blocking of the IGF-1R by JB-3 reduced the degree of neuroprotection by TES on the axotomized RGCs, indicating that TES activates an intrinsic retinal IGF-1 system that then rescues the axotomized RGCs (by IHC).

#### NES effect on inflammatory markers

The retinal protection of NES partially depends on its anti-inflammatory properties. Inflammation plays a major role in glaucoma progression. To determine whether TES impacted the inflammatory response, immunolabeling for CD3 was used to evaluate whether T cells had infiltrated the retina, and Microglia were immunolabeled with Iba1 [50]. After TES treatment, the number of CD3 + T cells and Iba1 + microglia cells notably decreased. The percent area fraction of inner retina that was labeled with Iba1 + microglia was significantly higher for control eyes compared to TES-treated eyes (by IHC). Similarly, Lin Fu et al. [49] demonstrated that after AOH injury, the protein expression of phosphorylated nuclear factor-κB-p65 (p-NFκB-p65) in the retina was upregulated 1.67-fold (by WB), the mRNA expression level of TNF-α was upregulated 2.18-fold (by RT-PCR) compared to normal retina, and Interleukin 6 (IL-6) increased at both gene and protein levels (by WB, RT-PCR). TES treatment inhibited the upregulation of these factors. Meanwhile, the expression level of cyclooxygenase-2 (COX-2) in TES-treated retina was 82.7% lower than that in the sham-treated retina (by WB), and the anti-inflammatory cytokine IL-10 was significantly increased (by WB, RT-PCR), which was associated with a suppression of microglia cell activation in TES-treated eyes.

In the normal retina [58], astrocytes and Müller cells were in a resting state, ramified microglia were distributed in a mosaic pattern, with very few amoeboid microglia, and very few Tumor Necrosis Factor-α (TNF-α) positive cells were detected. Following ONT, Müller cells, astrocytes, and microglia were activated, in which microglia underwent morphological changes, going from ramified to rod- or ameboid-shaped. TNF-α is a pro-inflammatory cytokine that is rapidly upregulated and promotes RGC death after optic nerve injury. TNF-α was colocalized with ameboid microglia, but not with rod microglia, astrocytes, or Müller cells, which indicates that ameboid microglia are the source of TNF-α after ONT. However, TES significantly decreased the expression of TNF-α from ameboid microglia (by IF and WB).

#### NES effect on retinal cells apoptosis

NES saves retinal nerve cells by influencing the expression of apoptotic factors. In the model of light damage [42], the upregulation of B-cell lymphoma-2 (Bcl-2) and the downregulation of BCL-2-associated X protein (Bax) in the retina after TES were related to their neuroprotective effects. Bcl-2 increased sharply from 2 h after TES, reaching its peak at 6–7 h, and remained elevated for 14 days. Bax was downregulated from post-TES 2 h (by qRT-PCR, WB). In which, Bcl-2 immunoreactivity appeared in the end feet and processes of Müller cells (by IHC). In MNU-induced RP mice [45], TES downregulated the expression levels of Bax and Calpain-2, Conversely, the expression level of Bcl-2 was upregulated after TES treatment, indicating that apoptotic-associated genes were involved in the TES-induced protective effects against MNU toxicity (by qRT-PCR).

In addition, Hanif et al. [40] revealed a significant upregulation of Caspase 3 expression after WES. Although caspase 3 is frequently associated with the process of cell death, it also contributes to cell survival in mildly stressful situations.

#### Other effects and mechanisms

Hanif et al. [40] found that glutamine synthetase (GS) expression appears to rise in response to ES therapy, which could lead to increased glutamate turnover rates and reduced susceptibility to glutamate excitotoxicity. Müller cells were where the majority of GS immunoreactivity was found in normal retinas [48]. The end-feet areas of Müller cells showed a small increase in GS immunoreactivity six hours after ischemia in the sham-stimulated retinas. The strongest immunoreactivity for GS was reached at the 24th hour as intense immunoreactivity moved from the inner limiting membrane to the outer limiting membrane. Next, it started to decline on day 7 and continued to do so until it reached nearly normal levels on day 14 (by IHC). In the ischemic rats model induced by H-IOP, the GS protein level in TES-treated retinas began to increase at 6 h after ischemia, peaked at 24 h, accounting for 322% of the normal retina, and decreased to near normal levels on the 14th day. Except for the 14th day after ischemia, the GS expression levels in TES-treated retinas were significantly higher than those in the control retinas at each time point.

Moreover, for correct neural signaling, energy homeostasis is crucial, and abnormalities in retinal and optic nerve metabolism have been seen in the glaucoma D2 model [50]. AMP-kinase (AMPK) is a key metabolic regulator of ATP availability. In the optic nerve and retina treated with TES, the pAMPK/AMPK ratio was significantly lower than in the control group, indicating that TES rescued ATP decreased (by WB).

## Discussion

### Summary of evidence

There is a great need for neuroprotective therapies for retinal disease. Ideal neuroprotective techniques increase the survival of neurons by maintaining their structure and function [28, 62]. The current study summarized the retinal protective effect of NES, a kind of relatively safe physical therapy, which was described in the literature as a neuroprotective technique capable of ameliorating the damage of RP, RD, H-IOP, ON trauma, and NAION. However, all of the abovementioned retinal disorders have the same pathological alterations, which are abnormalities in the structure and function of nerve cells or other retinal cell components that range from primary to tertiary neurons. NES frequently targets these underlying causes of many diseases and finally has a therapeutic impact, which has been confirmed by our current systematic review. The analyzed articles showed that different animal models of retinal diseases benefit from different types of NES treatment, both functionally and structurally.

NES improved retinal and visual function in assessments like electrophysiological analysis and functional testing, including raised ERG and VEP amplitudes, improved the average spatial frequency threshold ratio, increased the signal-to-noise ratio, and affected the connection networks between neurons in the primary visual cortex and prefrontal cortex. Besides, NES not only affects the retina and its related functions, but can also modulate neurons in the brain. Yu et al. [63] found that TES exerts antidepressant-like effects by improving neuroplasticity (including neurogenesis and synaptic plasticity) in the hippocampus and amygdala. Alzheimer's disease and aged mice with cognitive dysfunction also benefit from TES treatment [64].

The functional protections of NES are closely associated with the structural maintenance of retinal cellular components. NES saved the thickness of ONL (contains the nucleus of photoreceptors), the density of cones and rods, and the length of IS/OS, thus improving cell survival and protecting the degeneration damage of the photoreceptors. For RGC injury, NES preserved the cell density in the RGC layer and reduced cell death while increasing the viability of their axons. Besides, NES inhibits the activity of microglia, and promotes the differentiation potential of Müller cells by directly stimulating them.

**Fig. 2:**
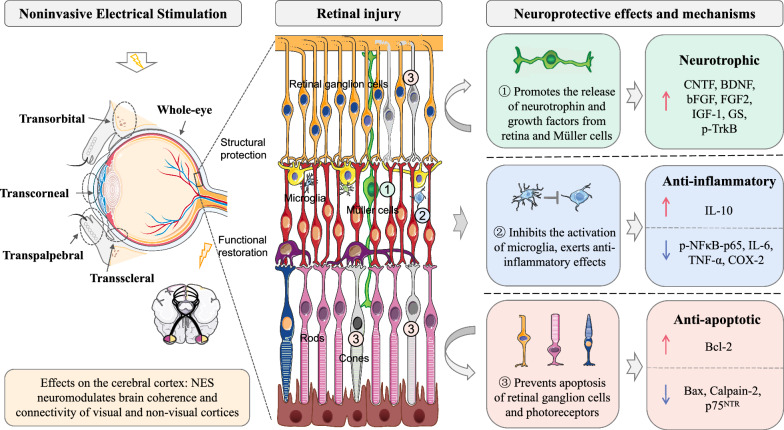
Main categories of noninvasive electrical stimulation (NES) and possible neuroprotective mechanisms underlying the effects. The cellular structure in the figure represents an enlarged image of the retina. Gray cells represent damaged cells in retinal diseases. It has been discovered that NES protects the structure and function of retinal ganglion cells and photoreceptors and has a positive effect on other cell components, including Müller cells, and microglia. The neuroprotective effect of NES involved a variety of mechanisms, including neuro-nutrition (No. ①, green), alleviation of inflammation (No. ②, blue), and inhibition of apoptosis (No. ③, pink). The red upward arrow represents upregulation, while the blue downward arrow represents downregulation. *CNTF* ciliary neurotrophic factor, *BDNF* brain-derived neurotrophic factor, *bFGF* basic fibroblast growth factor, *FGF-2* fibroblast growth factor 2, *IGF-1* insulin-like growth factor 1, *GS* glutamine synthetase, *p-TrkB* phosphorylated tyrosine kinase receptor B, *p-NFκB-p65* phosphorylated nuclear factor-κB-p65, *TNF-α* tumor necrosis factor-α, *IL-6* interleukin 6, *COX-2* cyclooxygenase-2, *IL-10* interleukin 10, *Bcl-2* B-cell lymphoma-2, *Bax* BCL-2-associated X protein

Studies of proteomics and gene expression profiling by Kanamoto et al. [65] and Willmann et al. [66] found that the neuroprotective effect of NES may involve a variety of mechanisms, it stimulates neurotrophic factors and cell survival pathways by modulating proteins and genes involved in cellular signaling, neuronal transmission, metabolism, and inflammation. The neuroprotective effect of NES that the current systematic review has demonstrated mainly involves the following aspects: (1) NES promotes the release of neurotrophin and growth factors from retina and Müller cells, including CNTF, BDNF, FGF-2, IGF-1, bFGF, and p-TrkB (p-TrkB is an indication of its activity and binding of BDNF). Specifically, NES increased the expression of BDNF and FGF-2 in the whole retina, and upregulated the levels of CNTF, BDNF, and IGF-1 in Müller cells. Similar increases in BDNF, IGF-1, and FGF-2 were found in electrically stimulated Müller cell cultures, and the release of the growth factor was likely due to stimulation of L-type voltage-dependent calcium channels [67–69]. NES-induced retinal production of bFGF contributes to protecting the survival of retinal cells while enhancing the proliferation and neurogenic potential of Müller cells. Furthermore, NES could attenuate glutamate-mediated excitotoxicity by increasing the level of GS in Müller cells. (2) NES inhibits the activation of microglia and exerts anti-inflammatory effects. The anti-inflammatory properties of NES are mainly achieved by downregulating p-NFκB-p65, TNF-α, IL-6, and COX-2, as well as upregulating the expression of cytokine IL-10. (3) NES prevents apoptosis of retinal ganglion cells and photoreceptors. It exerts an anti-apoptotic role by downregulating Bax, Calpain-2, and p75^NTR^, and upregulating Bcl-2. Additionally, NES improved energy homeostasis by reducing the pAMPK/AMPK ratio. The main categories and potential neuroprotective mechanisms of NES are shown in Fig. 2.

In summary, NES plays a neuroprotective role in the structure and function of the retina by promoting neuro-nutrition, reducing inflammation, and inhibiting cell apoptosis; almost no adverse effects in the included animal studies were reported. The results provide sufficient evidence for further clinical studies.

### NES as a potential clinical technique

#### Bringing NES therapy to clinic

For multiple retinal neurodegenerative diseases, NES therapy has been applied to clinical practice [70–72]. At present, there are 11 NES trials (including RP and RD) listed on clinicaltrials.gov [73]. There are also small sample clinical reports on NES treatment for AMD, and diseases mainly characterized by damage to RGCs (glaucoma, nonarteritic ischemic optic neuropathy, or traumatic optic neuropathy) [74–76]. These patient populations may all benefit from the treatment of NES. As one of the attractive candidates, rapidly translating NES into the clinic is a goal for the future. However, some issues need to be taken seriously.

Generally, starting NES therapy at the earliest stage of the disease will most effectively delay progression. In animal experiments, treatment can be initiated immediately after model building. However, in the clinical setting, visual loss is usually detected months to years after the onset of retinal disease, and the chance of significantly preventing visual loss is minimal when treatment is initiated in the middle or late stages of retinal disease. Several studies have used TES in patients with long-term visual loss due to retinal artery occlusion and reaped some benefits, but whether these results can be improved by early treatment or by an optimized stimulation paradigm is unclear [77, 78]. Thus, early screening and intervention for retinal disease may contribute to the clinical translation of NES. Besides, retinal diseases progress very slowly, the intended target of NES is to slow or halt the progression of the disease. To make measurable differences in the control group compared to the NES group, the study design requires following the subjects for a sufficient length of time, with careful consideration of the potential diversity of the patient population or disease status (subtype, stage), and a reasonable selection of appropriate outcome measures [28].

In the current neuroprotective strategies, the targeted survival pathway drugs (anti-apoptotic agents, growth factors) can be targeted to the retina to produce beneficial effects, but how to effectively deliver the intervention to the target tissue is a problem. NES, as a rehabilitation therapy, extensively regulates the retina by directly stimulating the eye and thus activating the endogenous repair mechanisms. Each of these strategies has the potential to be used in several different retinal diseases, and through specific or multiple pathways, resulting in increased retinal neuronal survival and preservation of visual function. However, there is a lack of studies combining two or more strategies, including matching ES with targeted survival drugs. Osaka et al. [59] reported the benefits of steroids or TES on anatomic changes and visual function in a rat model of nonarteritic ischemic optic neuropathy, and determined that the two treatments may be complementary, steroids are effective for reducing disc edema, while TES is effective for preserving RGCs function and structure. Unfortunately, they did not test the two treatments together, but this study still gives us inspiration: study designs targeting multiple combinations of neuroprotective strategies need to be considered in the future.

#### Optimization of parameters

The types and parameters of NES are particularly important for the implementation of clinical practice [79, 80]. The existence of an optimal stimulation protocol that is generally applicable to all subjective species is unrealistic [81]. The stimulation parameters, such as the pulse duration, current intensity, stimulation frequency, and repetition frequency, should be adjusted reasonably and varied according to the pathological type and subjective species. In rats, Takeshi Morimoto et al. [57] reported that the optimal neuroprotective parameters for TES were pulse duration of 1 and 2 ms/phase, current intensity of 100 and 200 μA, stimulation frequency of 1, 5, and 20 Hz, and duration at least 30 min. Interestingly, TES dose-dependently exerts neuroprotective effects in retinal diseases. The protective effect of electrical stimulation at 50 μA, 100 μA, 200 μA, and 300 μA was enhanced as the current intensity increased. Similar results have been confirmed in human trials. Alfred Stett et al. [82] found that loss of visual field area in patients with RP was significantly reduced in treated eyes compared to untreated eyes by regular use of TES in a dose-dependent manner, TES treatment is most effective above 0.8–1.0 mA (5 ms/phase, 20 Hz). For the patients who have a branch retinal artery occlusion, the intensity to elicit a phosphene ranged between 0.5–0.9 mA (20 Hz, 30 min), and the phosphene was perceived in both the peripheral and central visual fields [78]. This does not mean that the current intensity can be increased without hesitation. As the current intensity reaches the threshold, its neuroprotective effect may decrease. When the TES was increased to 100 μA and 200 μA, there was a significant increase in the density to 85.4% and 80.0%, respectively, of intact retinas, however, an increase of TES to 300 μA and 500 μA resulted in a decrease in the mean RGC densities to 70.0% and 64.5%, respectively, of intact retinas [57].

Hanif et al. [40] and Ying-qin Ni et al. [42] reported different timings of gene expression after ES, which is crucial for selecting the repetition frequency. The gene expression of BDNF and FGF-2 increases occur quickly, by 1 h post-WES, and are back to normal by 24 h post-WES [40]. After TES, the mRNA levels of BDNF peaked on the 3rd day, CNTF peaked on the 7th day, and both of them decreased at 14 days [42]. Although they chose different stimulation routes and parameters, the results can inspire us. Compared to daily TES, daily WES stimulation may produce larger protective effects on maintaining gene expression changes and possibly further protecting the structure and function of retina, on the contrary, once or twice a week TES may be better, which may be enlightening for the selection of clinical repetition frequency. Similarly, in terms of pulse duration, stimulation frequency, it is not necessarily the case that the higher the parameter, the better the effect. In clinical practice, the stimulation dose must be adjusted to the individual tolerance level that patients can withstand, and the limits of the safe current density on the ocular surface must also be taken into account.

#### Complications and safety profile

As a retinal neuroprotective strategy, NES’ s goal is to provide the optimal therapeutic dose for the retina while minimizing side effects. The most frequently used route of NES in currently included animal experiments was TES (17 of 21 studies). TES only contacts the cornea, which greatly reduces the risk of serious complications compared to retinal implants. There is only one report on complications of TES in the articles we included [42]. After a TES with parameters of 400 μA and 50 Hz, rats exhibited corneal epithelial proliferation and retinal perforation, which may be attributed to high charge density stimulation causing some of the current density not to be properly dissipated at the retina and resulting in injury. Therefore, prolonged use of high degrees of stimulation is not advised. In clinical trials, only a small number of complications of TES have been reported [83–85]. An article that included over 1000 patients reported the following local side effects for TES: foreign body sensation, dry eye syndrome (reported in ~ 3% and 15% of cases, respectively), and transient superficial keratitis (reported in ~ 5% of cases) [1]. Overall, the safety of TES therapy is positive, since such complications are easily addressable, although not to be ignored if repeated stimulation is needed for optimal results.

For most retinal diseases, the treatment process may take several years, the impact of long-term TES treatment is not yet known. According to Yang et al. [86], although TES did not affect tear production, it increased the possibility of ocular surface injury by reducing mucin (MUC) 4 expression and conjunctival secretion of MUC5AC in vivo. While literature often fails to distinguish between which electrodes and which application mode were used when assessing safety. In most TES animal experiments, contact lens electrodes were applied to the cornea, in clinical TES studies, DTL electrodes were used, these differences may have an impact on the assessment of safety. Unlike TES, TpES increased tear production but did not cause corneal fluorescein staining. The electrical resistance from the orbital skin to the TpES was lower than that from the cornea to the retina in the TES. Thus, as another safe and effective ES method for treating retinal neurodegeneration, further conducting large-scale clinical trials and in-depth animal experiments to elucidate the efficacy and mechanism of TpES is another future direction. In addition, there have been no reports of complications with other types of ES, one reason may be that there are fewer studies (4 of 21 studies) compared to TES, the same issues confront clinical research as well.

### Limitations

Certain restrictions on this systematic study should be noted. (1) The SYRCLE's evaluation revealed that the included studies' general quality is moderate. To increase the validity and rigor of the investigations, it is recommended that emphasis be given to the full reporting of random sequence generation, allocation concealment, random outcome assessment, and the use of blinding in the future. (2) The studies included in this systematic review were inconsistent in key areas such as the models used to represent retinal disorders and the route, parameters used for NES therapy, which added to the research's heterogeneity. Statistics for grouping were not taken into account due to the methodological diversity among studies. As a result, no meta-analyses were carried out using the available data. (3) Since only studies published in English were included in this systematic review, there may be differences in language and regional literature that were missed, which could have an impact on the extrapolation of the systematic review's findings.

## Conclusion

In this systematic review, NES demonstrated neurotrophic, anti-inflammatory, and anti-apoptotic capabilities as a neuroprotective method adopted in retinal illnesses with high security. These findings backed up the idea that NES has the potential to be a successful therapy for the treatment of retinal disorders. To evaluate the effectiveness of NES in a therapeutic environment, however, well-designed randomized controlled clinical trials are required.

## Data Availability

All data generated or analyzed during this study are included in this published article.

## Abbreviations

AMD: Age-related macular degeneration
AMPK: AMP-kinase
AOH: Acute ocular hypertension
Bcl-2: B-cell lymphoma-2
BDNF: Brain-derived neurotrophic factor
bFGF: Basic fibroblast growth factor
CNTF: Ciliary neurotrophic factor
COX-2: Cyclooxygenase-2
CSLO: Confocal scanning laser ophthalmoscope
EEG: Electroencephalography
ERG: Electroretinogram
ES: Electrical stimulation
FG: Fluorogold
FGF-2: Fibroblast growth factor 2
GCL: Ganglion cell layer
GS: Glutamine synthetase
HE: Hematoxylin–eosin
H-IOP: High-intraocular pressure injury
ICON: In vivo confocal neuroimaging
IF: Immunohistofluorescence
IGF-1: Insulin-like growth factor 1
IHC: Immunohistochemistry
IL-6: Interleukin 6
LGN: Lateral geniculate nucleus
MEA: Multi-electrode array
MeSH: Medical subject headings
MNU: *N*-Methyl-*N*-nitrosourea
MUC: Mucin
NAION: Nonarteritic ischemic optic neuropathy
NES: Noninvasive electrical stimulation
OKT: Optokinetic tracking
ONC: Optic nerve crush
ONL: Outer nuclear layer
ONT: Optic nerve transection
OPs: Oscillatory potentials
PAC: Phase amplitude coupling
p-NFκB-p65: Phosphorylated nuclear factor-κB-p65
PRISMA: The Preferred Reporting Items for Systematic Reviews and Meta-Analyses
qRT-PCR: Quantitative real-time PCR
RB: Rose Bengal
RCS: Royal College of Surgeons
RD: Retinal degeneration
RGCs: Retinal ganglion cells
RoB: Risk of bias
RP: Retinitis pigmentosa
SC: Superior colliculus
SD: Sprague-Dawley
SNR: Signal-to-noise ratio
STR: Scotopic threshold responses
SYRCLE: Systematic Review Centre for Laboratory Animal Experimentation
TES: Transcorneal electrical stimulation
TNF-α: Tumor necrosis factor-α
TpES: Transpalpebral Electrical Stimulation
TrkB: Tyrosine Kinase Receptor B
TsES: Transscleral electrical stimulation
TUNEL: Terminal-deoxynucleotidyl transferase-mediated nick end labeling
VEP: Visually evoked potentials
VIST: Vision-test
WB: Western Blot
WES: Whole-eye electrical stimulation
